# 2D Nb_2_C MXene-enhanced hierarchical hydrogel for efficient solar-driven water evaporation

**DOI:** 10.1039/d5ra01735a

**Published:** 2025-05-15

**Authors:** Guangyao Zhang, Deqi Fan, Zongze Li, Yingying Li, Yi Lu

**Affiliations:** a Jiangsu Co-Innovation Center of Efficient Processing and Utilization of Forest Resources, International Innovation Center for Forest Chemicals and Materials, College of Science, Nanjing Forestry University Nanjing 210037 China yilu@njfu.edu.cn; b Jiangsu Co-Innovation Center of Efficient Processing and Utilization of Forest Resources, International Innovation Center for Forest Chemicals and Materials, College of Chemical Engineering, Nanjing Forestry University Nanjing 210037 China

## Abstract

Solar-driven interfacial evaporation has emerged as a sustainable solution to address global freshwater scarcity by converting solar energy into thermal energy for efficient water purification. To achieve rapid and energy-efficient steam generation, the development of advanced photothermal materials with optimized light absorption and water activation remains critical. Here, we present a three-dimensional polyvinyl alcohol/chitosan/Nb_2_C MXene (PCN) hydrogel engineered for high-performance solar evaporation. The composite integrates Nb_2_C MXene nanosheets into a hydrophilic polymer matrix through hydrogen bonding and electrostatic interactions, forming interconnected microchannels that enable broadband solar absorption (93% across 300–2500 nm) *via* MXene's plasmonic effects and light-trapping architecture. The synergistic combination of rapid water transport and weakened hydrogen bonding within the hydrated network significantly reduces the evaporation enthalpy to 1426 J g^−1^. This design achieves an exceptional evaporation rate of 2.72 kg m^−2^ h^−1^ and a solar-to-vapor conversion efficiency of 93.2% under 1 sun irradiation, surpassing conventional hydrophilic polymer-based systems. The hydrogel's hierarchical porous structure facilitates effective thermal localization and sustains stable evaporation across varying solar intensities (1–5 sun), demonstrating adaptability for scalable applications. This work provides a rational strategy to design MXene-enhanced hydrogels for practical solar desalination and wastewater purification technologies.

## Abstract 2

Nb_2_C MXene hydrogel achieves 93% solar absorption *via* plasmonic/light-trapping synergy, lowers evaporation enthalpy to 1426 J g^−1^, enabling 2.72 kg m^−2^ h^−1^ rate under 1 sun for scalable solar desalination.

## Graphical abstract

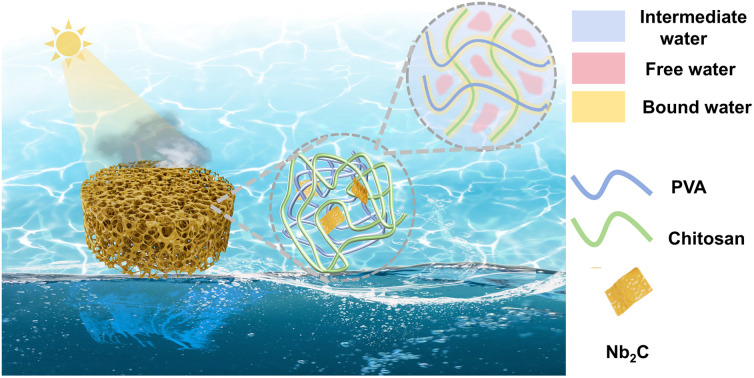

## 1. Introduction

With societal development and population growth, freshwater scarcity has emerged as a critical global challenge.^1^ Seawater desalination, particularly through solar-driven technologies, has become a sustainable solution to address water resource shortages.^2,3^ Conventional methods such as reverse osmosis and electrodialysis suffer from high energy consumption and costs, limiting their application in resource-scarce regions.^4,5^ Solar-driven interfacial evaporation technology has gained prominence as a promising alternative, utilizing abundant sunlight to produce freshwater with minimal environmental impact.^6,7^ This approach relies on photothermal materials capable of efficiently converting solar energy into localized heat at the liquid–gas interface, enabling rapid vapor generation.^7,8^ Consequently, the development of high-performance solar photothermal materials capable of harnessing full-spectrum solar energy remains imperative.

To achieve high water evaporation rates and solar thermal conversion efficiency, extensive efforts have focused on optimizing photothermal materials. Carbon-based materials (*e.g.*, graphene, carbon nanotubes) and plasmonic metals (*e.g.*, gold/silver nanoparticles) exhibit strong spectral absorption and conversion capabilities.^9–13^ However, their inherent hydrophobicity often restricts water transport and evaporation rates, with most reported carbon-based evaporators underperforming compared to hydrophilic polymer hydrogels. For instance, polyvinyl alcohol (PVA)-based systems integrated with photothermal components have demonstrated enhanced water supply and reduced evaporation enthalpy.^2,7,14,15^ MXene, a two-dimensional transition metal carbide, stands out due to its exceptional broadband light absorption (>90% across 300–2500 nm) and photothermal conversion efficiency, attributed to localized surface plasmon resonance and high electrical conductivity.^16–18^ The incorporation of MXene not only enhances photothermal performance but also improves hydrophilicity. He *et al.* developed a sulfonated polyacrylamide (PAM) hydrogel with hydrophilic sulfonic groups, achieving a reduced evaporation enthalpy (1187 J g^−1^) and a high evaporation rate of 2.50 kg m^−2^ h^−1^ under 1 sun irradiation *via* a polypyrrole-loaded PAM-melamine foam evaporator.^19^ Chen *et al.* engineered a vertically structured PVA hydrogel evaporator with MXene@TiO_2_@g-C_3_N_4_ (MTG) heterojunctions, which enhanced light absorption through multiple reflections and reduced evaporation enthalpy by modulating polymer–water interactions, while enabling photocatalytic degradation.^20^ Despite these advancements, balancing high evaporation efficiency, scalable fabrication, and long-term stability remains challenging.

In this study, we developed a poly(vinyl alcohol) (PVA)-chitosan (CS) dual-network hydrogel through a covalent crosslinking strategy, innovatively incorporating oxidation-resistant Nb_2_C MXene as a photothermal core. Compared to conventional single-network systems, the synergistic hydrogen bonding and electrostatic interactions between PVA and CS enabled the construction of a three-dimensional interconnected porous architecture with balanced mechanical robustness and dynamic tunability.^21,22^ Notably, the protonated amino groups (–NH_3_^+^) on CS chains formed stable ionic interactions with oxygen-terminated (–O) surfaces of Nb_2_C MXene. This *in situ* self-assembly mechanism not only achieved uniform dispersion of MXene nanosheets but also addressed the chronic oxidation issues of traditional Ti_3_C_2_ materials in aqueous environments.^23–26^ The hydrogel network regulated interfacial water states through competitive hydrogen bonding interactions, effectively reducing the evaporation enthalpy to 1426 J g^−1^. Meanwhile, the broad-spectrum solar absorption (93% across 300–2500 nm) of Nb_2_C synergized with light-trapping effects from the hierarchically porous structure, collectively enhancing photothermal conversion efficiency.^22,27,28^ Under 1-sun irradiation, the hydrogel demonstrated an exceptional evaporation rate of 2.72 kg m^−2^ h^−1^ with 93.2% energy conversion efficiency, achieved without requiring Janus configurations or external auxiliaries. Compared with previously reported Ti_3_C_2_-based composites, this design advances solar desalination technology through dual innovations in intrinsic material stability and synergistic structural reinforcement, presenting a promising strategy for sustainable water purification.

## 2. Experimental section

### 2.1 Chemicals

Polyvinyl alcohol (PVA, alcoholysis degree: 87.0–89.0%) was purchased from Macklin Biochemical Co., Ltd. Lithium fluoride (LiF, 99.9%), glutaraldehyde (50% aqueous solution), and chitosan (<200 mPa s) were obtained from Aladdin. Dimethyl sulfoxide (DMSO, AR), anhydrous ethanol (AR), acetic acid (99.50%), and hydrochloric acid (HCl, 36–38%) were supplied by Shanghai Biochemical Reagent Co., Ltd. Nb_2_AlC powder was procured from Laizhou Kaixi Ceramic Materials Co., Ltd. All reagents were used without further purification.

### 2.2 Preparation of few-layer Nb_2_C MXene

First, 0.08 mol of LiF was added to 20 mL of 12 M HCl and stirred at 60 °C for 30 min to ensure complete dissolution. Subsequently, 0.5 g of Nb_2_AlC was gradually dispersed into the mixture. Then, the reaction was sealed in a polytetrafluoroethylene (PTFE) container and maintained at 60 °C for 9 days to complete the etching process. The resulting product was repeatedly washed with 2 M HCl and ultrapure water *via* centrifugation until a neutral pH (∼7) was achieved, yielding multilayer Nb_2_C. The obtained multilayer Nb_2_C powder (0.1 g) was mixed with 15 mL of dimethyl sulfoxide (DMSO) under Ar protection and stirred continuously for 108 h, with periodic ultrasonication to promote intercalation of the Nb_2_C layers. The mixture was then washed thoroughly with ethanol (AR) and ultrapure water to obtain few-layer Nb_2_C.

### 2.3 Fabrication of PCN hydrogel

A 10 wt% PVA solution was prepared by dissolving 10 g of PVA in 90 mL of 80 °C deionized water under vigorous stirring. Separately, 2 g of chitosan (CS) was dissolved in 48 mL of a 2 wt% acetic acid solution to form a 2 wt% CS solution. The PVA and CS solutions (10 mL each) were mixed to form a precursor solution. Few-layer Nb_2_C powder (20 mg) was uniformly dispersed into the mixture under stirring. Glutaraldehyde (0.5 mL, 5 wt%) as a crosslinking agent and HCl (0.7 mL, 1 M) as a catalyst were then slowly dripped into the solution. Gelation was completed within 6 h. The hydrogel was immersed in deionized water to remove residual chemicals, surface-dried with filter paper, frozen in liquid nitrogen, and thawed in water at 40 °C. This freeze-thaw cycle was repeated 10 times to obtain the final PCN hydrogel. PVA and PVA/CS hydrogels were fabricated using the same strategy.

### 2.4 Characterization

The morphology and microstructure of few-layer Nb_2_C MXene powder were examined using a JSM-7600F scanning electron microscope (SEM). The hydrogel's structure was analyzed *via* a Thermo Scientific Prisma environmental SEM. X-ray diffraction (XRD, Ultima IV, Hitachi) with Cu Kα radiation was employed to characterize crystallinity. Surface hydrophilicity was assessed using a contact angle meter (DSA100, KRÜSS, Germany). Solar absorption spectra were acquired using a UV-vis–NIR spectrophotometer (Lambda 950, PerkinElmer) equipped with an integrating sphere. Fourier transform infrared spectroscopy (FTIR, VERTEX 80 V, Bruker, Germany) were performed to measure and analyze the phase of the composites. Dynamic mechanical analysis (DMA) was conducted using a micro-infrared coupled rheometer (MAS60, Anton Paar GmbH) with oscillatory strain and frequency sweep modes.

### 2.5 Solar evaporation performance evaluation

Evaporation experiments were conducted using a solar simulator (CEL–PE300L–3A, CEAULIGHT) with an AM1.5 optical filter. Light intensity (1, 3, and 5 kW m^−2^) was calibrated using a photometer (CEL–NP2000–2, CEAULIGHT). Mass loss of evaporated water was monitored in real time *via* an electronic balance (FA2004, ±0.0001 g accuracy). Surface temperature profiles were recorded using an infrared thermal camera (Fotric 325L). Tests were performed at ambient conditions (25 °C, 40% relative humidity).

## 3. Results and discussion

**Fig. 1:**
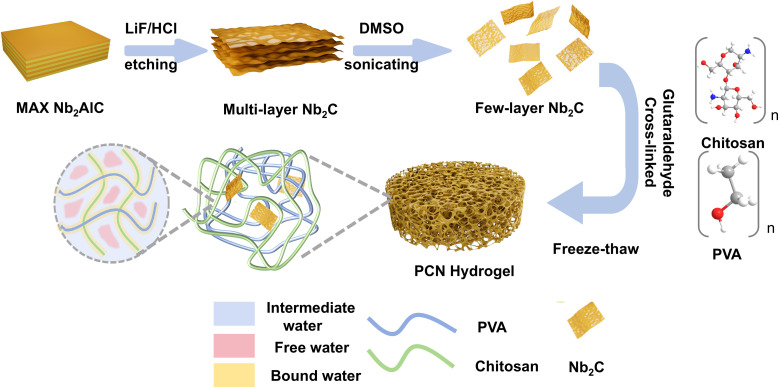
Schematic illustration of the fabrication process and working mechanism of the 3D hydrogel solar evaporator based on few-layer Nb_2_C MXene.

In order to enhance the photothermal conversion capability of MXene within hydrogels, it is essential to cultivate a smooth and complete nanosheet structure, which is characterized by a substantial specific surface area. Fig. 1 delineates the sequence of steps involved in the synthesis of Nb_2_C (MXene) few-layer nanosheets, along with their utilization in the fabrication of PVA/CS (chitosan)/Nb_2_C (PCN) hydrogels. The preparation of few-layer Nb_2_C nanosheets involved HCl/LiF etching (a safe alternative to direct HF usage), a method that circumvents the hazards associated with hydrofluoric acid. In this process, the Al atomic layers in Nb_2_AlC are selectively etched to obtain multilayer Nb_2_C, followed by delamination to yield few-layer Nb_2_C nanosheets. The photothermal evaporation performance of the resulting few-layer Nb_2_C nanosheets exhibited concentration-dependent enhancement, with optimal performance achieved at 20 mg loading (1 mg mL^−1^ in hydrogel matrix). Further details of the synthesis method can be found in the Experimental section. The gel matrix demonstrates exceptional thermal insulation, rapid water transport through hierarchical channels, and reduced evaporation enthalpy *via* hydrogen-bond regulation, collectively enabling efficient solar-driven vapor generation.

**Fig. 2:**
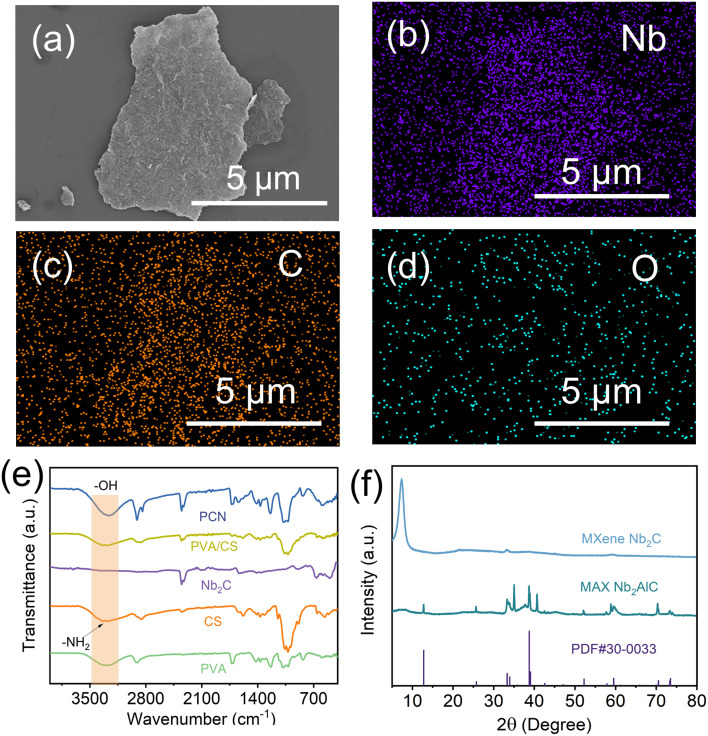
(a) Scanning electron microscopy (SEM) image of few-layer Nb_2_C MXene; (b–d) corresponding EDS elemental mapping of few-layer Nb_2_C; (e) FTIR spectra of PVA, chitosan, Nb_2_C, PVA/CS, and PCN; (f) XRD pattern of few-layer Nb_2_C MXene.

Scanning electron microscopy (SEM) imaging was used to observe the preparation of Nb_2_C MXene nanosheets. As demonstrated in Fig. 2, the two-dimensional nanosheet morphology of few-layer Nb_2_C MXene provides an enlarged specific surface area, which enhances light-to-heat conversion through multi-photon absorption. Energy-dispersive spectroscopy (EDS) mapping (Fig. 2b–d) confirms homogeneous distribution of Nb, C, and F elements on the Nb_2_C MXene surface. X-ray diffraction (XRD) analysis was conducted to verify the successful synthesis (Fig. 2f). The pristine Nb_2_AlC exhibited diffraction peaks matching standard PDF#30-0033. Post-etching, the characteristic (104) peak at 39.0° disappeared, confirming complete Al atmolayer removal. The (002) peak shifted from 12.79° to 7.3°, corresponding to interlayer spacing expansion from 0.69 nm to 1.21 nm. This structural evolution facilitates enhanced water molecule intercalation and photothermal conversion efficiency, consistent with established MXene modification mechanisms.^29–32^ The chemical interactions within the Nb_2_C/PVA/CS composite evaporator were systematically characterized by Fourier transform infrared (FTIR) spectroscopy (Fig. 2e). In the spectrum of pure PVA, the broad absorption band spanning 3200–3500 cm^−1^ arises from hydroxyl (–OH) stretching vibrations, while the distinct peaks at 1070 cm^−1^ and 2900 cm^−1^ correspond to C–O and C–H stretching modes, respectively.^33^ Chitosan exhibits characteristic vibrational signatures including a –C–O bending mode at 1070 cm^−1^, a primary amine deformation band at 1590 cm^−1^, and a –N–H stretching vibration spanning 3200–3400 cm^−1^.^34^ Notably, the 1590 cm^−1^ amine vibration serves as a critical spectral fingerprint for distinguishing chitosan from PVA. Following glutaraldehyde crosslinking, the composite hydrogel demonstrates complete suppression of the N–H stretching signal (3200–3400 cm^−1^), confirming the formation of Schiff base linkages between chitosan's amino groups and aldehyde functionalities.^35,36^

**Fig. 3:**
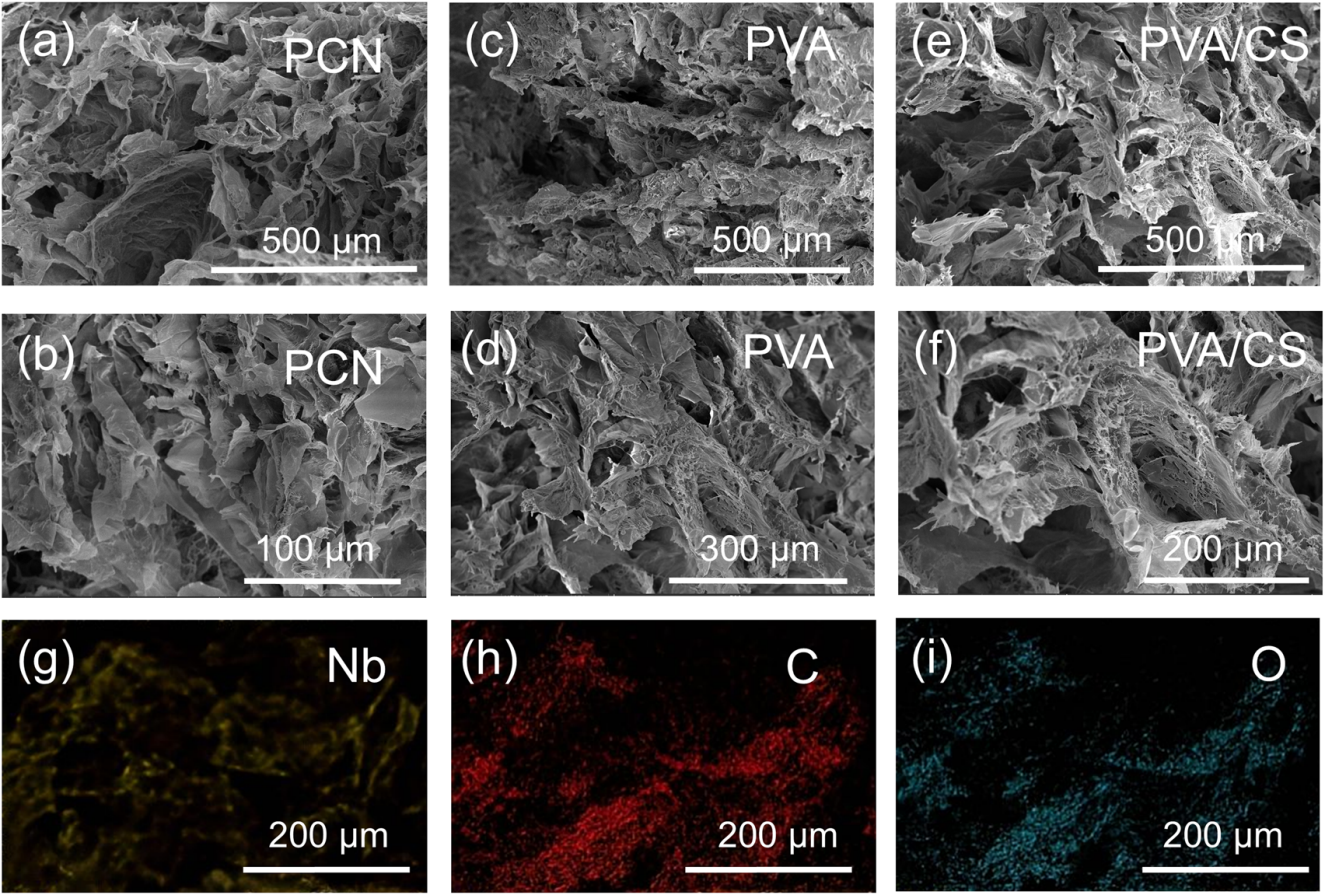
(a and b) Environmental SEM images of PCN hydrogel; (c and d) PVA hydrogel; (e and f) PVA/CS hydrogel; (g–i) EDS elemental mapping of PCN hydrogel.

The etched Nb_2_C MXene powders were then utilized in the preparation of PCN hydrogels. The microstructures of the synthesized PVA hydrogels, PVA/CS hydrogels, and PCN hydrogels are exhibited in Fig. 3. All hydrogels possess a micron-scale porous structure, characterized by interconnected channels and thin walls. These macroporous capillary channels are conducive to rapid internal water transport. The Nb_2_C surface is rich in oxygen-containing groups (*e.g.*, –OH, –O, and –F terminations), which enhance broadband solar absorption, particularly in the near-infrared region (NIR, 800–1500 nm).^37^

Moreover, the –OH groups of Nb_2_C form an extensive hydrogen-bond network with the –OH groups of PVA through hydrogen bonding and electrostatic interactions with the amino groups (–NH_2_) of chitosan or the hydroxyl groups (–OH) of PVA. Chitosan molecules are adsorbed on the Nb_2_C nanosheet surfaces through electrostatic interactions (attractive force: –NH_3_^+^ ↔ –O^−^), neutralizing their negative surface charge, reducing nanosheet agglomeration, and improving uniform dispersion.^38^ Concurrently, the –NH_2_ of chitosan and the –O groups of Nb_2_C form ionic bridges, thereby enhancing the material's mechanical strength.^39^ As illustrated by the scanning electron microscope images (Fig. 3a and b), the PCN hydrogel exhibits a more regular internal pore distribution than the PVA and PVA/CS hydrogels. The EDS spectrum mapping (Fig. 3g–i) reveals that the Nb_2_C nanosheets are uniformly dispersed within the hydrogel, exhibiting minimal agglomeration, which is conducive to efficient photothermal evaporation.

**Fig. 4:**
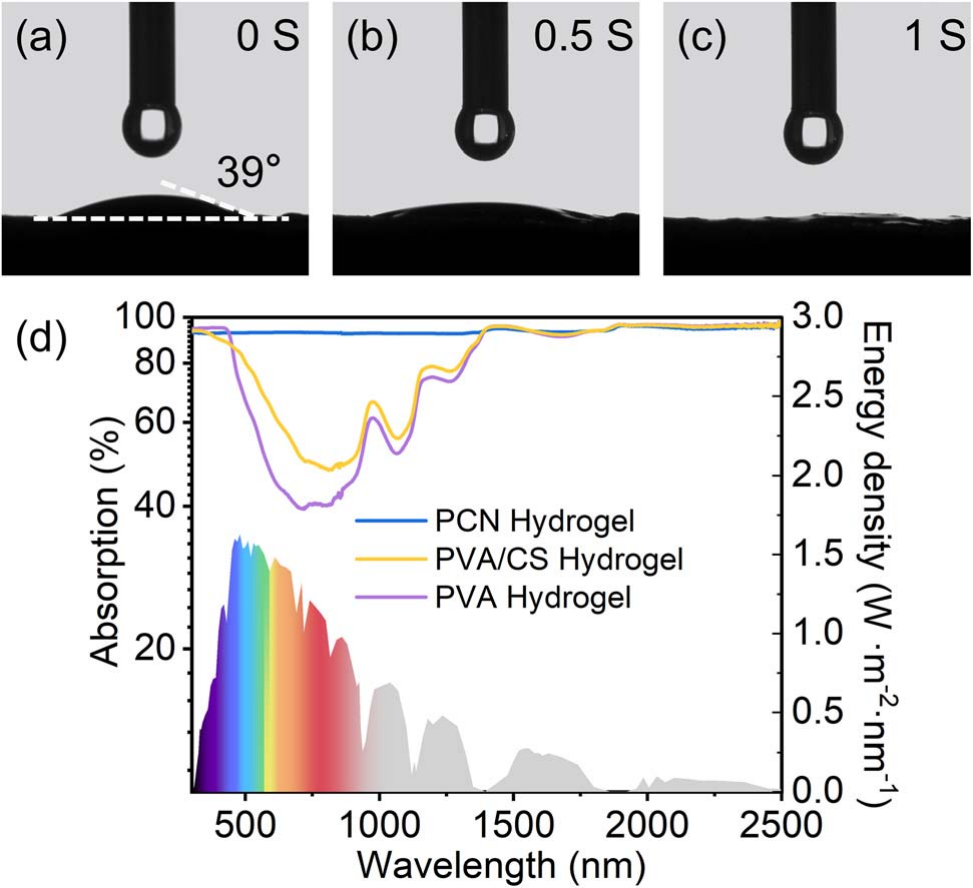
(a–c) Time-lapse snapshots of hydrophilicity test on PCN surface; (d) solar absorption spectra of PCN, PVA, and PVA/CS hydrogels in 300–2500 nm wavelength range.

The hydrophilic character of the material serves as a critical determinant for efficient solar-driven water evaporation to quantitatively evaluate the hydration dynamics of the hydrogel evaporator surface, water contact angle measurements were systematically performed. The dynamic wetting process of deionized water droplets was captured using a high-speed contact angle analyzer, revealing complete surface hydration within one second for the PCN hydrogel (Fig. 4a–c). This rapid wettability transition enables continuous water supply through synergistic capillary action and upward pumping effects, effectively sustaining evaporation rates under prolonged operation. The engineered hydrogel further demonstrates dual photothermal advantages. Its interconnected porous network promotes broadband light absorption by inducing multi-photon scattering events that extend the effective optical path length. These structural and compositional features collectively ensure efficient vapor generation while maintaining mechanical stability during cyclic.

The solar absorptivity of various hydrogels was systematically investigated through UV-vis–NIR spectroscopy across the 300–2500 nm spectral range. The wavelength-dependent absorption efficiency was calculated *via* the equation:1
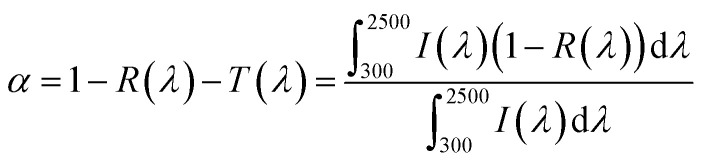
where *R*(*λ*) denotes the spectral reflectance and *I*(*λ*) represents the solar irradiance distribution integrated over the AM 1.5 G solar spectrum.^40–42^ Benchmark measurements revealed distinct absorption characteristics: the pristine PVA hydrogel demonstrated limited solar harvesting capability with 62% (Fig. 4d), while chitosan incorporation in PVA/CS composites elevated the value to 70%. Progressive MXene integration induced substantial enhancement in photon capture, with PCN hydrogels showing concentration-dependent absorptivity improvement (Fig. 4d). Notably, the system of 20 mg MXene loading (1 mg mL^−1^) achieved near-perfect absorption 93%, beyond which no significant enhancement in absorptivity was observed, indicating saturation of photon harvesting sites.

**Fig. 5:**
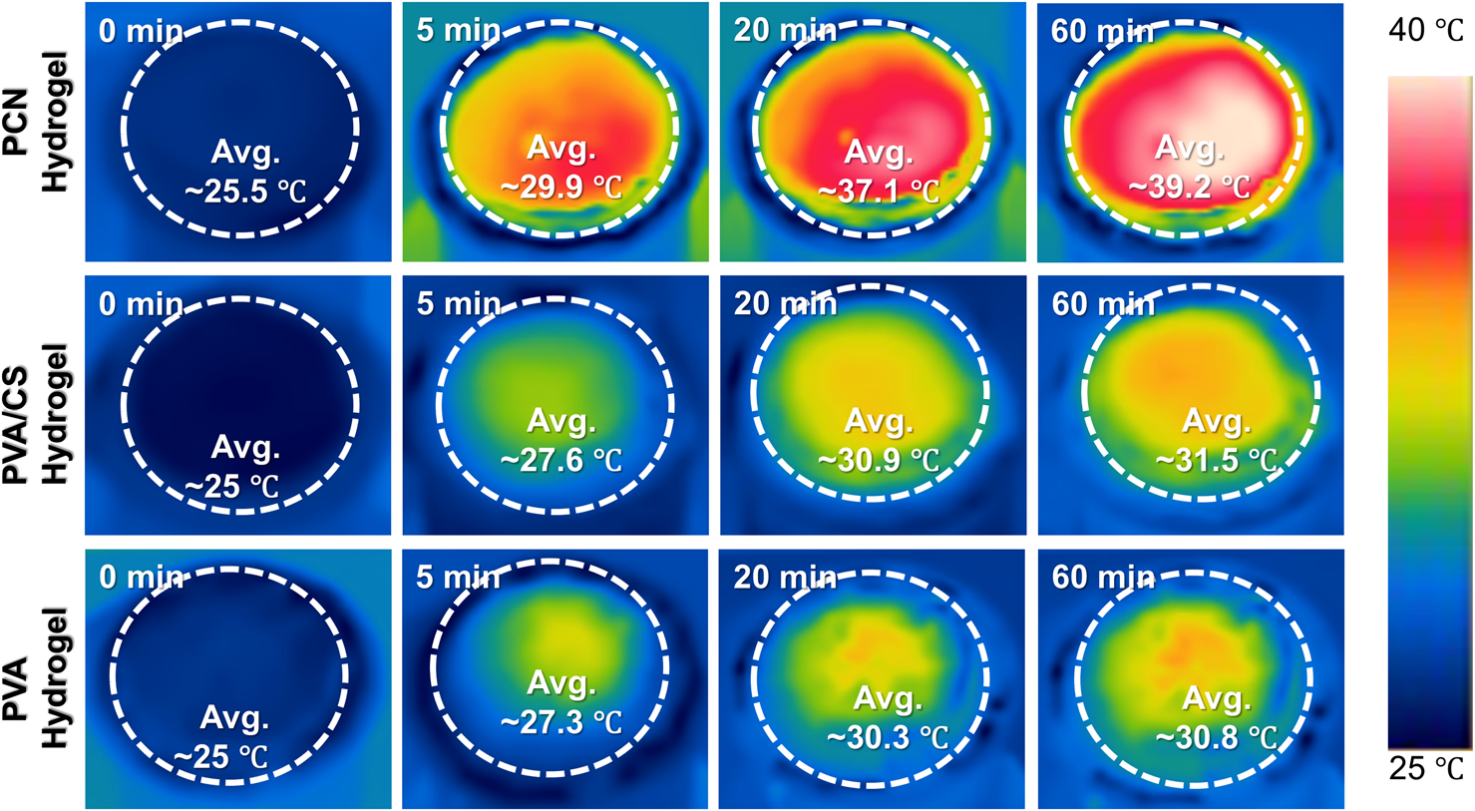
Infrared thermal images of PCN, PVA, and PVA/CS hydrogels under 1 sun irradiation.

**Fig. 6:**
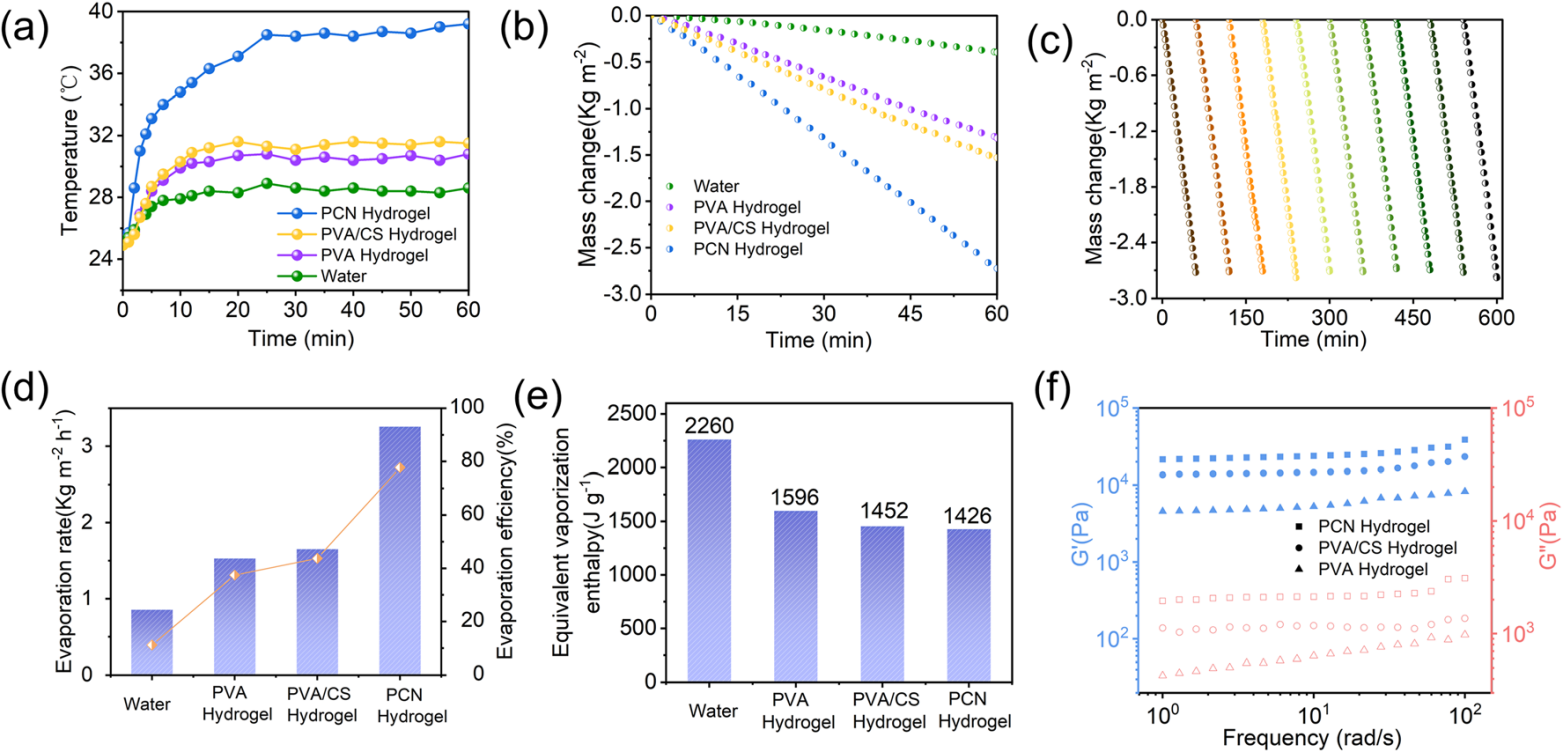
(a) Temperature evolution profiles; (b) water mass loss; (c) the long-term stability test of solar evaporation of PCN hydrogel. (d) evaporation rate with solar-thermal conversion efficiency; (e) equivalent evaporation enthalpy of PCN, PVA, and PVA/CS hydrogels under 1 sun illumination; (f) dynamic mechanical analysis of different hydrogels (DMA).

To investigate the photothermal conversion properties of the samples, infrared thermography was employed to monitor surface temperature changes during evaporation (Fig. 5 and 6a). Under 1 sun irradiation (1 kW m^−2^), the equilibrium surface temperatures reached 39.2 °C for PCN hydrogel, compared to 30.8 °C and 31.5 °C for PVA and PVA/CS hydrogels, respectively. The composite hydrogel demonstrates excellent hydrophilicity and efficient light absorption, facilitating photothermal evaporation. The evaporation performance was evaluated by measuring mass loss under 1 sun irradiation (1 kW m^−2^).

**Fig. 7:**
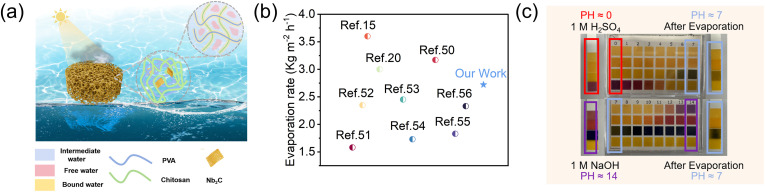
(a) Schematic diagram of PCN hydrogel for seawater desalination with internal mechanism; (b) evaporation rates comparison between PCN evaporator and other hydrophilic polymer materials; (c) pH values before and after treating strongly acidic/alkaline wastewater.

The PCN hydrogel exhibited the highest evaporation rate of 2.72 kg m^−2^ h^−1^ (Fig. 6b and c), exceeding PVA hydrogel (1.31 kg m^−2^ h^−1^) and PVA/CS hydrogel (1.53 kg m^−2^ h^−1^). Dark-field experiments showed the evaporation enthalpy of hydrophilic hydrogels was lower than pure water, with further reduction in CS-loaded hydrogels. This reduction is attributed to increased evaporation surface area from porous microstructures and hydrophilic groups (–OH, –NH_2_) in PVA, CS, and Nb2C. These groups regulate water states *via* non-covalent interactions, enhancing intermediate water content in cross-linked networks, thereby lowering evaporation enthalpy (Fig. 6d and 7a).^15,40,43^ In addition, we conducted cyclic stability tests on the PCN hydrogel (Fig. 6c) by performing 10 consecutive evaporation cycles (0–60 mins per cycle) under 1-sun irradiation. No evident performance decay was observed in water evaporation efficiency, demonstrating its potential for industrial applications. As shown in Fig. 6f, dynamic mechanical analysis (DMA) revealed that the elastic modulus (*G*′) of all hydrogels consistently exceeded the viscous modulus (*G*′′) across the tested frequency range, exhibiting typical gel-like characteristics. Notably, the PVA/CS hydrogel demonstrated enhanced mechanical properties compared to the PVA hydrogel due to the formation of a dual-crosslinked network through chitosan incorporation and freeze-thaw cycles. The PCN hydrogel exhibited the highest elastic modulus, confirming that MXene incorporation effectively reinforced the mechanical strength.^44,45^

The energy conversion efficiency of the evaporator was calculated using the measured evaporation rate and equivalent evaporation enthalpy, where *η* represents the energy conversion efficiency, *v*_e_ is the net evaporation rate (dark evaporation subtracted), *C*_opt_ is the incident solar flux (1 kW m^−2^), and *H* denotes the sample-specific evaporation enthalpy.^46–49^2
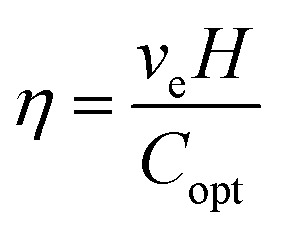


The calculated energy conversion efficiencies reached 93.1% for the PCN hydrogel, 43.7% for PVA, and 47.2% for PVA/CS hydrogel. For comparison, a hydrogel evaporator with increased Nb_2_C MXene content was tested, but showed no significant improvement in water transport capacity, evaporation rate, or energy conversion efficiency. Comparative analysis with literature-reported hydrophilic polymer hydrogel evaporators confirmed the superior evaporation performance of the PCN hydrogel, exceeding most counterparts as shown in Fig. 7b.^15,20,50–56^ Solar-driven purification of strongly acidic (1 M H_2_SO_4_) and alkaline (1 M NaOH) wastewater was conducted using the PCN hydrogel. The treated water exhibited near-neutral pH (6.8–7.2), meeting standard requirements for domestic water use (Fig. 7c). These results demonstrate the PCN hydrogel evaporator's potential for industrial wastewater purification applications.

**Fig. 8:**
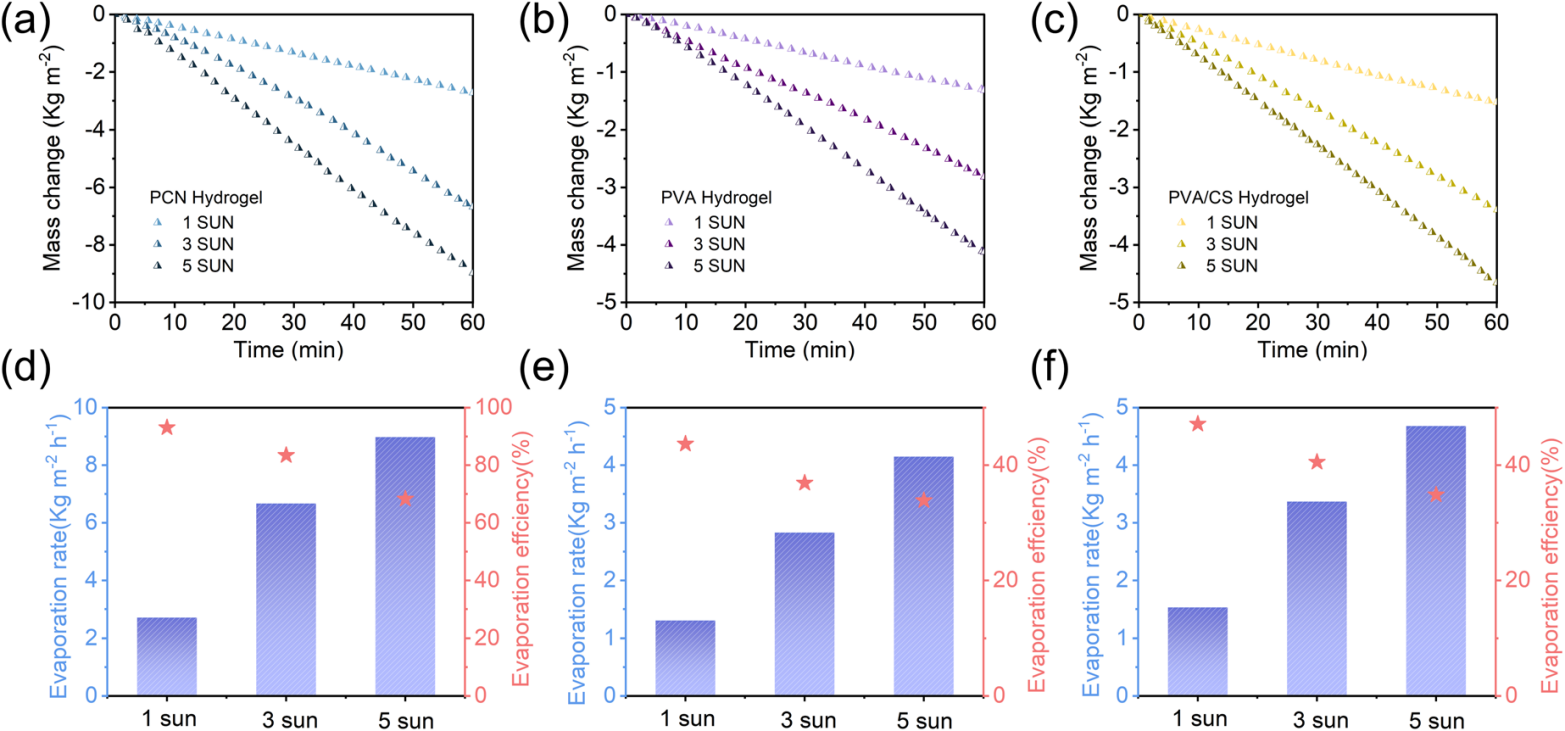
(a–c) Water mass loss, (d–f) corresponding evaporation rates with efficiencies of PCN, PVA, and PVA/CS hydrogels under different solar irradiation intensities (1–5 sun).

A systematic investigation of evaporation behavior under gradient solar irradiance (Fig. 8) revealed a strong correlation between photothermal conversion efficiency and light intensity. Experimental data demonstrate that as irradiance increases from 1 sun to 5 sun, all hydrogel evaporators exhibit linear mass loss kinetics across illumination conditions, indicating stable evaporation rates. The PCN hydrogel achieved the most efficient performance, with evaporation rates linearly increasing from 2.72 kg m^−2^ h^−1^ (1 sun) to 6.67 kg m^−2^ h^−1^ (3 sun) and 8.98 kg m^−2^ h^−1^ (5 sun), corresponding to energy conversion efficiencies of 93.08%, 83.44%, and 68.28%, respectively. This enhancement originates from effective thermal localization enabled by the three-dimensional porous network structure. In contrast, PVA and PVA/CS hydrogels followed typical linear responses (1 sun: 1.31/1.53 kg m^−2^ h^−1^; 5 sun: 4.15/4.68 kg m^−2^ h^−1^), maintaining relatively low conversion efficiencies due to their higher equivalent evaporation enthalpy and restricted light-harvesting capacity. This work demonstrates enhanced irradiance adaptability through integrated structural design, providing insights for industrial-scale solar distillation system development.

## 4. Conclusion

In summary, a Nb_2_C MXene-enhanced hydrogel evaporator was fabricated through hydrogen bonding and electrostatic assembly. The composite structure achieves 93% solar absorption *via* MXene's plasmonic effects and hierarchical pores, while modified water interactions lower evaporation enthalpy to 1426 J g^−1^. Under 1 sun, the system demonstrates a stable evaporation rate of 2.72 kg m^−2^ h^−1^ and 93.2% solar evaporation efficiency. Moreover, it exhibits good stability in harsh chemical conditions (1 M H_2_SO_4_/NaOH), showing potential for seawater desalination and industrial wastewater treatment. This work provides a practical strategy for designing MXene-based hydrogels for solar water purification.

## Data availability

The data are available from the corresponding author on reasonable request.

## Author contributions

Guangyao Zhang: conceptualization, writing – original draft. Deqi Fan: writing – original draft, investigation, methodology. Zongze Li: investigation, methodology, formal analysis. Yingying Li: methodology, data curation. Yi Lu: writing – review & editing, funding acquisition.

## Conflicts of interest

The authors declare that they have no known competing financial interests or personal relationships that could have appeared to influence the work reported in this paper.
